# Extensive Testing and Public Health Interventions for the Control of COVID-19 in the Republic of Cyprus between March and May 2020

**DOI:** 10.3390/jcm9113598

**Published:** 2020-11-08

**Authors:** Annalisa Quattrocchi, Ioannis Mamais, Constantinos Tsioutis, Eirini Christaki, Costas Constantinou, Maria Koliou, Zoi-Dorothea Pana, Valentinos Silvestros, Fani Theophanous, Christos Haralambous, Androulla Stylianou, Sotiroula Sotiriou, Maria Athanasiadou, Theopisti Kyprianou, Anna Demetriou, Christiana A. Demetriou, Ourania Kolokotroni, Ioanna Gregoriou, Niki Paphitou, George Panos, Leontios Kostrikis, Peter Karayiannis, Georgios Petrikkos, Petros Agathangelou, George Mixides, Georgios Siakallis, Linos Hadjihannas, Lakis Palazis, Anna Vavlitou, Chrystalla Matsentidou-Timiliotou, Dimitris Koukios, Tonia Adamidi, Frangiskos Frangopoulos, Elizabeth Constantinou, Georgios Nikolopoulos

**Affiliations:** 1 Department of Primary Care and Population Health, University of Nicosia Medical School, 1700 Nicosia, Cyprus; quattrocchi.a@unic.ac.cy (A.Q.); demetriou.chri@unic.ac.cy (C.A.D.); kolokotroni.o@unic.ac.cy (O.K.); 2 School of Sciences, European University, 1516 Nicosia, Cyprus; i.mamais@euc.ac.cy; 3 School of Medicine, European University, 1516 Nicosia, Cyprus; k.tsioutis@euc.ac.cy (C.T.); Z.Pana@euc.ac.cy (Z.-D.P.); gpetrikkos@gmail.com (G.P.); 4 Medical School, University of Cyprus, 1678 Nicosia, Cyprus; christaki.eirini@ucy.ac.cy (E.C.); koliou-mazeri.maria@ucy.ac.cy (M.K.); panos.george@ucy.ac.cy (G.P.); 5 Nicosia General Hospital, 1678 Nicosia, Cyprus; csconstandin@yahoo.gr (C.C.); hadjihannas_l@hotmail.com (L.H.); aimika@cytanet.com.cy (L.P.); vavlitouanna@hotmail.com (A.V.); tadamide@mphs.moh.gov.cy (T.A.); frangopoulos@yahoo.com (F.F.); 6 Unit for Surveillance and Control of Communicable Diseases, Ministry of Health, 1448 Nicosia, Cyprus; silval87@hotmail.com (V.S.); ftheophanous@mphs.moh.gov.cy (F.T.); charalambous@mphs.moh.gov.cy (C.H.); astylianou@mphs.moh.gov.cy (A.S.); ssoteriou@mphs.moh.gov.cy (S.S.); igregoriou@mphs.moh.gov.cy (I.G.); EConstantinou@moh.gov.cy (E.C.); 7 Health Monitoring Unit, Ministry of Health, 1448 Nicosia, Cyprus; MAthanasiadou@moh.gov.cy (M.A.); tkyprianou@moh.gov.cy (T.K.); ademetriou@moh.gov.cy (A.D.); 8 American Medical Center, 1311 Nicosia, Cyprus; elpnik@gmail.com (N.P.); gmix@msn.com (G.M.); 9 Department of Internal Medicine, Division of Infectious Diseases, Patras University General Hospital, Medical School, University of Patras, 265 04 Patras, Greece; 10 Department of Biological Sciences, University of Cyprus, 1678 Nicosia, Cyprus; lkostrik@ucy.ac.cy; 11 Department of Basic and Clinical Sciences, University of Nicosia Medical School, 1700 Nicosia, Cyprus; karayiannis.p@unic.ac.cy (P.K.); siakallis.g@med.unic.ac.cy (G.S.); 12 Heart and Medical Center, Nicosia Heart Institute, 2410 Nicosia, Cyprus; Paga@cytanet.com.cy; 13 Larnaca General Hospital, 6301 Larnaca, Cyprus; 14 Limassol General Hospital, 3304 Limassol, Cyprus; hristalla@cytanet.com.cy (C.M.-T.); dkoukios@gmail.com (D.K.)

**Keywords:** surveillance, COVID-19, pandemic, contact tracing, containment measures, Cyprus

## Abstract

Coronavirus disease 2019 (COVID-19) has significantly affected the well-being of individuals worldwide. We herein describe the epidemiology of COVID-19 in the Republic of Cyprus during the first epidemic wave (9 March–3 May 2020). We analyzed surveillance data from laboratory-confirmed cases, including targeted testing and population screening. Statistical analyses included logistic regression. During the surveillance period, 64,136 tests (7322.3 per 100,000) were performed, 873 COVID-19 cases were diagnosed, and 20 deaths were reported (2.3%). Health-care workers (HCWs) represented 21.4% of cases. Overall, 19.1% of cases received hospital care and 3.7% required admission to Intensive Care Units. Male sex (adjusted Odds Ratio (aOR): 3.04; 95% Confidence Interval (CI): 1.97–4.69), increasing age (aOR: 1.56; 95%CI: 1.36–1.79), symptoms at diagnosis (aOR: 6.05; 95%CI: 3.18–11.50), and underlying health conditions (aOR: 2.08; 95%CI: 1.31–3.31) were associated with hospitalization. For recovered cases, the median time from first to last second negative test was 21 days. Overall, 119 primary cases reported 616 close contacts, yielding a pooled secondary attack rate of 12% (95%CI: 9.6–14.8%). Three population-based screening projects, and two projects targeting employees and HCWs, involving 25,496 people, revealed 60 positive individuals (0.2%). Early implementation of interventions with targeted and expanded testing facilitated prompt outbreak control on the island.

## 1. Introduction

Coronavirus disease 2019 (COVID-19), the respiratory illness caused by the severe acute respiratory syndrome coronavirus 2 (SARS-CoV-2), has affected more than 32.7 million people worldwide by September 27th, 2020 [1]. First reported in Wuhan, China it was declared a public health emergency of international concern and later, a pandemic, by the World Health Organization (WHO) [2].

The Republic of Cyprus is an island country in the Southeast Mediterranean, with a population of approximately 875,900 (government-controlled area). The first COVID-19 cases in Cyprus, detected on 9 March 2020, were two people with recent travel history abroad, from Italy and the United Kingdom, respectively. Since then, the authorities instituted a bundle of containment interventions, including social distancing measures, school and university closures, and travel and movement restrictions. In addition, active contact tracing, targeted testing, and reallocation of healthcare resources were implemented [3].

The aims of these analyses are to describe the epidemiological characteristics of COVID-19 cases in the Republic of Cyprus, to report the results of extensive testing and contact tracing, and to outline the course of the epidemic in association with the implemented measures, which led to significant control of SARS-CoV-2 transmission. Secondary outcomes of interest are also the description of the characteristics of all COVID-19 cases, those hospitalized and those admitted in the Intensive Care Units (ICU) as well as the characteristics of deceased and recovered cases. Last, the estimation of pooled and individual secondary attack rates was another secondary outcome of interest.

## 2. Material and Methods

### 2.1. Data Source and Surveillance System

During the COVID-19 pandemic, the Unit for Surveillance and Control of Communicable Diseases (USCCD), within the Department of Medical and Public Health Services (MPHS) of the Ministry of Health (MoH), was responsible for surveillance and public health interventions. In addition, a Scientific Advisory Committee consisting of national experts was created to advise directly the health authorities regarding the management of the pandemic.

A total of 17 laboratories (five public and 12 private) routinely performed molecular diagnostic testing for SARS-CoV-2 during the surveillance period. Epidemiological data and characteristics of individuals with SARS-CoV-2 infection, detected from 9 March to 3 May 2020 (i.e., age, sex, place of residence, occupation, date of sampling and of laboratory-confirmed diagnosis, origin of infection, number of close contacts, symptoms, underlying health conditions, hospital and ICU admissions, intubation status, date and cause of death, date and result of follow-up testing) were reported to an electronic platform of USCCD. Data were collected as part of surveillance activities and public health emergency interventions, in accordance with the legal framework mentioned above [4]. All data were anonymized. 

The submission of the current manuscript was approved by the Cyprus National Bioethics Committee.

### 2.2. Definitions

A suspect case was initially defined as an individual with symptoms (general malaise/ weakness, and/or temperature >37.3 °C, and/or dry cough, and/or muscle aches) and close contact with a laboratory-confirmed case or history of travel abroad (affected areas) over the previous 14 days. On March 23rd, the epidemiological criteria were removed from the suspect case definition [5], as part of active case finding activities and in accordance with European recommendations [6].

A confirmed case was defined as a person with a respiratory swab (nasopharynx and/or pharynx) positive for SARS-CoV-2 through real-time reverse-transcription polymerase chain (rRT-PCR) assay, with or without symptoms [7].

Cases were categorized as imported if they had a history of travel from an affected area in the 14 days before disease onset. Primary cases were individuals who tested positive for SARS-CoV-2 and had the earliest onset date in the Republic of Cyprus and no travel history from affected areas. Secondary cases were those with confirmed close contact with a known primary case. Tertiary cases had confirmed close contact with a known secondary case. Primary, secondary, and tertiary cases were defined as locally acquired cases [8].

A close contact of a confirmed case was defined as a person with face-to-face contact in any setting, within two meters for more than 15 minutes or, with physical or unprotected contact in a closed environment with a COVID-19 case [9].

A health-care worker (HCW) was defined based on occupation and not on place of exposure. HCWs were defined as all health-care professionals, allied health workers (i.e., physicians, nurses, and other health occupations), and auxiliary health workers [10].

Asymptomatic cases were considered to be recovered/cured if they had two consecutive negative tests at least 14 days after diagnosis with at least a 24-h interval between the two tests. For symptomatic or for hospitalized cases, COVID-19 was considered cured after the resolution of symptoms and two negative tests as described above [11].

Secondary attack rate (SAR) was defined as the proportion of infections among susceptible people who were close contacts of confirmed cases [12].

### 2.3. Public Health Interventions and Measures

Public health interventions and measures aiming to suppress viral transmission were deployed in four phases:

Period 1 (10–14 March): school and university closures, cancellation of large public gatherings (>75 persons); Period 2 (15–23 March): access to the Republic of Cyprus allowed only for specific persons and after SARS-CoV-2 testing, travelers from countries reporting community transmission had to remain under medical observation for 14 days, travelers from countries reporting local transmission or imported cases had to remain in self-isolation for 14 days, entertainment areas (e.g., malls, theaters, hotels) closures, only 1 person per 8 square meters in public service areas allowed; Period 3 (24–30 March): restrictions in construction sites, closure of the majority of retail services; Period 4 (31 March–3 May): incoming flights suspended except chartered flights for special cases and repatriated Cypriot citizens, compulsory 14-day quarantine for all inbound travelers, inter-city travel prohibited and inner-city movements restricted, night curfew, all social gatherings prohibited.

### 2.4. Contact Tracing and Testing Strategy

Contact tracing was performed by USCCD of the MPHS, through phone calls with confirmed cases, according to WHO guidance [13]. All close contacts of confirmed cases were instructed to self-isolate for 14 days after last contact with the index case and were tested within 24–72 hours for SARS-CoV-2, irrespective of symptoms.

During the surveillance period, alongside with testing of suspect cases and close contacts of confirmed cases, further active case finding activities were performed as follows: 4–9 April: Population screening of an age-stratified sample of residents in two municipalities (hereafter named A and B), where the 14-day cumulative notification rate was more than double the total 14-day cumulative notification rate in the Republic of Cyprus (aggregate data reported to the MoH); 10–30 April: Targeted testing of employees in the public domain, retail food and beverage services, customer services, of personnel of National Guard, and in nursing homes; 17 April–1 May: Sample testing of HCWs in public hospitals (aggregate data reported to the MoH); 23–26 April: Population screening (random, age- and geographic-stratified sampling) throughout the Republic of Cyprus. For the last population screening, probability sampling proportional to size was performed, based on district of residence and age. The population was divided into four districts out of five and five age groups (0–19, 20–39, 40–59, 60–79, 80+ years). One district (where Municipality A was previously sampled) and one Municipality (i.e., B) were excluded. The sampling frame was the Health Insurance Organization list and individuals were contacted through landline phone calls.

### 2.5. Statistical Analysis

We conducted complete case-analysis. Mean and standard deviation (SD) and median and interquartile range (IQR) were used to describe continuous data, while frequencies and percentages were presented for categorical variables. *t*-test, ANOVA, and chi-squared tests or Fisher test (where appropriate) were used in univariable analyses. Age-specific notification rate by sex was calculated using last national population data [14], and age-adjusted case fatality ratio (CFR) by sex was calculated using the 2013 European standard population [15].

Logistic regression analysis was performed to identify independent risk factors for hospitalization. Due to the small sample size, multivariable analysis was performed only to compare the characteristics of hospitalized and non-hospitalized cases. We used the principle of parsimony, by choosing the fewest number of variables (e.g., sex, age, presence of symptoms, and suffering of underlying conditions), which also helps avoid collinearity. We used a forward stepwise approach and variables were entered in the multivariable logistic regression model one-by-one. We used the likelihood ratio (LR) test to compare the fit among nested models and we selected the model with the lowest log likelihood value if the LR test was significant, as the final model. Adjusted OR (aOR) and 95% Confidence Intervals (CI) were calculated. A *p*-value < 0.05 was considered to be statistically significant.

Pooled SAR with its 95%CI was calculated by dividing the number of confirmed secondary cases by the total number of close contacts of the primary or imported cases, excluding those who did not report any close contacts [16]. Individual SARs were also estimated for each case that reported close contacts along with their mean (±SD).

All statistical analyses were conducted using STATA^®^ version 14 (StataCorp., College Station, TX, USA).

## 3. Results

### 3.1. Characteristics of Cases

**Figure 1:**
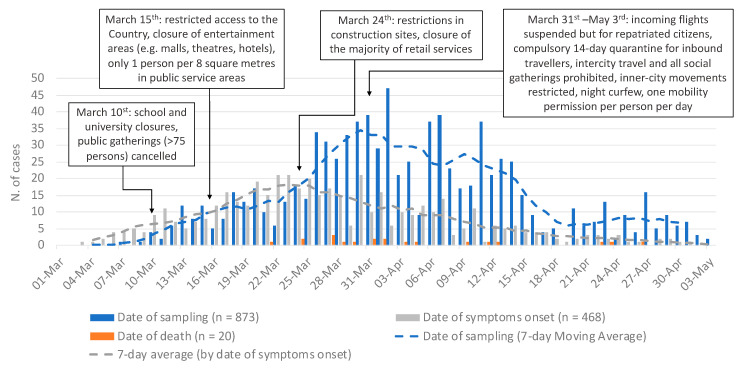
Number of laboratory-confirmed COVID-19-cases by date of sampling (blue), 7-day moving average by date of sampling (dash blue line), date of symptoms onset (gray), 7-day moving average by date of symptoms onset (dash gray line) and date of death (orange), and public health interventions implemented, Cyprus 1 March–3 May 2020.

As of 3 May 2020, 873 laboratory-confirmed COVID-19 cases had been reported. The cumulative notification rate was 99.7 per 100,000 population (95%CI: 93.3–106.5), and the 14-day cumulative notification rate peaked on 9 April at 49.3 per 100,000 population (Supplementary Figure S1). Table 1 reports the characteristics of cases and Figure 1 shows the number of cases by date of sampling, symptom onset and death, with details on public health interventions and control measures undertaken. Origin of infection was available for 83.5% of cases, of which 82.9% were locally acquired (Table 1). Cases were equally distributed between males and females. Most cases were aged 18–59 years (69.4%), followed by those aged ≥60 years (24.7%), and children/adolescents <18 years old (5.8%). Supplementary Table S1 reports the age-specific notification rate by sex. Overall, mean and median age were 45.9 and 46 years (IQR: 32–59 years), respectively. Based on occupation information, 21.4% were HCWs, of which 50.3% nurses, 21.9% auxiliary staff, 20.9% physicians, and 7% other (Table 1).

Clinical information at diagnosis was available for 98.4% of cases; 69.2% reported at least one symptom and 30.8% were asymptomatic at diagnosis. The most common symptoms (among symptomatic individuals) were cough (51%), fever (46.5%), and myalgia (32.7%). Furthermore, 27.9% reported three or more symptoms. Asymptomatic cases were mainly males (55.9% vs. female: 44.2%; *p* = 0.03), and younger than those who had symptoms (mean age: asymptomatic individuals 42.5 ± 19.0 vs. symptomatic individuals 47.3 ± 18.4 years; *p* < 0.001).

Nearly one-fifth were current smokers (19.2%). Information on underlying health conditions was available for 87.6% of cases, among which 41.2% had underlying health conditions and 12.8% had two or more conditions. Hypertension (16.4%), diabetes (8.9%), and heart disease (excluding hypertension) (8%) were the most frequently reported comorbidities. Mean age of people with underlying health conditions was statistically higher than those without (56.1 ± 18.0 vs. 39.1 ± 16.7 years; *p* < 0.001). No gender differences were reported between cases with and without comorbidities.

### 3.2. Characteristics of Hospitalized Cases

Almost one-fifth of all confirmed cases received hospital care (19.1%) (Table 2). The mean and median time from symptom onset to hospitalization was 7.8 and 6 days (IQR: 4–10), respectively. Hospitalized cases were significantly more males (65.9% vs. 34.1%; *p* < 0.001) and older (mean age: hospitalized 59.5 ± 18.3 vs. not hospitalized 42.7 ± 17.4 years; *p* < 0.001). More than two-thirds of hospitalized cases had underlying health conditions (69.5%).

Multivariable analysis showed that male sex (aOR: 3.04; 95%CI: 1.97–4.69), increasing age (aOR: 1.56; 95%CI: 1.36–1.79), presence of symptoms at diagnosis (aOR: 6.05; 95%CI: 3.18–11.50), and presence of underlying health conditions (aOR: 2.08; 95%CI: 1.31–3.31) were associated with hospitalization (Table 2). The overall mean hospital length of stay (LOS) was 9.4 ± 7.3 days (median 7; IQR 4–13 days). No gender differences were observed for LOS. However, there were significant differences in mean LOS by age group (*p* = 0.03). By May 3rd, more than two-thirds (72.5%) of patients had been discharged alive.

### 3.3. Intensive Care Unit Admission

Overall, 3.7% of cases required ICU admission and 3.1% were intubated, representing 19.2% and 16.2% of all hospitalized cases, respectively. ICU cases were significantly more males (71.9% vs. 28.1%; *p* = 0.012), with higher mean age compared to those not admitted to ICU (63.2 ± 13.8 vs. 45.3 ± 18.6 years; *p* < 0.001). The overall mean LOS in ICU was 15.8 ± 11.4 days (median 10.5; IQR 8–27 days). The mean ICU LOS for those alive (either still in ICU until May 3rd or discharged from ICU) was significantly higher than those who died in ICU (19.1 ± 11.9 vs. 10.8 ± 9.0 days; *p* = 0.04). The maximum number of COVID-19 patients in ICU at any given day was 15 (1.7 per 100,000).

### 3.4. Characteristics of Patients Who Died

Twenty COVID-19 patients died during the surveillance period (Table 1). The overall mortality rate was 2.3 per 100,000 and the CFR was 2.3%. The mean and median time from day of sampling to death were 10 and 7 days (IQR 3.5–15 days), respectively. CFR was higher in males than in females (3.7% vs. 0.9%; *p* = 0.011); and the age-adjusted mortality rate (per 100,000 population) was 4.7 in men and 1.2 in women. Among patients 0–39 years no deaths occurred, and CFR significantly increased from <1% in cases aged 40–59 years, to 5.4% in the age group 60–69 years and up to 16% in cases aged ≥80 years (*p* < 0.001). The mean age of those who died was higher than those who survived (73.1 ± 10.2 vs. 45.3±18.4 years; *p* < 0.001). CFR was higher among those with comorbidities (5.4% vs. 0.7%; *p* < 0.001). Notably, all deceased patients were hospitalized and died in hospital; hospital LOS did not statistically differ among those alive and those who died (9.2 ± 7.0 vs. 10.8 ± 9.1 days; *p* > 0.05). CFR was higher among cases admitted to ICU than in those not admitted to ICU (40.6% vs. 0.8%; *p* < 0.001) and among intubated compared with patients not intubated (37% vs. 1.2%; *p* < 0.001). Of note, among the 20 deaths recorded, COVID-19 was reported as the main underlying cause of death in 68%; thus, the COVID-19-specific CFR was 1.7%.

### 3.5. Characteristics of Recovered/Cured Cases

As of May 3rd, among 853 laboratory-confirmed COVID-19 cases alive, information on follow-up testing was available for 83.1% of cases. Overall, 55.6% cases had two consecutive negative tests after diagnosis. The median time from first to last negative test was 21 days (IQR 18–27). No significant differences were reported among recovered/cured and not recovered/cured cases with regard to sex, age, symptoms at diagnosis, comorbidities, and hospitalization, but for smoking status, sore throat, and anosmia (Supplementary Table S2).

### 3.6. Active Case Finding

**Figure 2:**
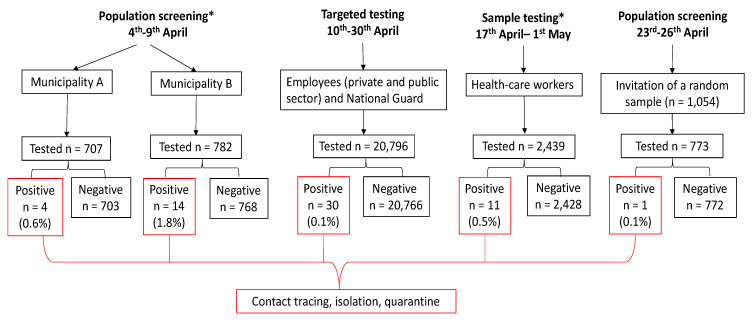
Outline of testing activities, Republic of Cyprus 9 March–3 May 2020. * aggregate data reported to the Ministry of Health.

By 3 May, a total of 64,136 diagnostic tests (7322.3 per 100,000) had been performed (Figure 2).

During the surveillance period, three population screening activities were conducted. Two were within a cluster screening initiative in early April in two municipalities. In Municipality A (a town of approximately 33,000 population), 707 individuals were tested, of which four were found positive (0.6%); in Municipality B (a town of approximately 19,000 population), out of 782 tests performed, 14 (1.8%) tested positive (Figure 2).

During the period 23–26 April 2020, of 1054 invitations for random population screening throughout the Republic of Cyprus (apart from municipalities A and B), 773 were tested (Figure 2). Mean and median age of participants were 40 ± 18.2 and 39 years (IQR: 28–53), respectively, and 53% were males. One person tested positive (0.13%), a 26-year-old female, who reported cough onset three days before testing. As a result of contact tracing, five close contacts were identified and tested; of these two were found positive (30 April).

Between 10–30 April 2020, 20,795 employees were tested (Figure 2). Mean and median age were 43 ± 11.8 and 43 years (IQR 34–52). Overall, 30 persons tested positive (0.14%). Table 3 shows the distribution of persons screened and those positive by sex and age group. Of note, no cases were detected among employees aged <20 or ≥65 years. From 17 April to 1 May, 2349 tests were performed among HCWs in public hospitals and 0.5% were positive for SARS-CoV-2 (Figure 2).

### 3.7. Secondary Attack Rate

Overall, 119 primary cases with 616 reported close contacts (mean number of close contacts: 5.2 ± 6.1) were identified. The pooled SAR was 12% (95%CI: 9.6–14.8%) and the mean individual SAR was 16.5% (±32.9%). Notably, two cases infected seven or more individuals. The first was a person who infected five individuals in the community and two in hospital. The second was an imported case who infected 10 relatives and one non-family member.

## 4. Discussion

The current report provides a detailed description of the first 9 weeks of the COVID-19 pandemic in the Republic of Cyprus and of the public health interventions that helped control SARS-CoV-2 transmission. An important feature of the public health approach was active case finding through continuous, extensive testing and contact tracing, which facilitated prompt detection and analysis of the characteristics of people with COVID-19 in the Republic of Cyprus. The testing rate in the Republic of Cyprus was among the highest in the European region during this period [17].

Early implementation of interventions in the Republic of Cyprus, consisting of school and university closures, cancellation of public gatherings, inter-city travel prohibitions, travel bans, and 14-day quarantine for incoming travelers, and mandatory isolation of cases and their close contacts, significantly decreased community transmission. Enhanced contact tracing significantly reduces the time spent in the community of a case during the period of infectiousness [18]. Also, limiting daily contacts through strict social restrictions are key strategies to reduce community transmission in population-based modeling studies [18,19]. Although it is hard to assess the benefits of school closures and transmissibility dynamics in children, particularly in low prevalence settings, proactive school closure has been proven beneficial to delay epidemic progression in pandemic influenza [20].

Among COVID-19 cases in the Republic of Cyprus, the most frequently reported symptoms and comorbidities are in line with most clinical reports from other countries [21,22]. In addition to increasing age, these were also the factors associated with need for hospital care, confirming the findings of prediction scores for severe disease [23,24]. Older age and male predominance of hospitalized patients, as well as a time interval of approximately 7 days between symptom onset and hospitalization, are similar to data published elsewhere [22]. Anosmia, a characteristic finding reported in COVID-19 patients, was reported in 16% of our series, showing the variable presentation of the disease and the importance of establishing all possible clinical manifestations. It is worth noting that as high as one third of our cases were asymptomatic upon detection, highlighting the importance of contact tracing and extended testing for early detection and isolation of contacts [25,26]. Although we did not determine the proportion of these patients who might have subsequently developed symptoms, the rate of asymptomatic infection in our case series falls within previously reported ranges [25,26].

As shown in other cohorts [27], people who died in the Republic of Cyprus were older and most of them with underlying health conditions. On 3 May, CFR was 2.3% and mortality rate 2.3 per 100,000 population, which is on the lower tier between European countries [28]. We believe that early public health interventions, along with healthcare system reinforcements, have significantly contributed to low CFR.

HCWs seem to be disproportionately affected by COVID-19. Similar to rates reported in the United Sates (19%) [29], 21.4% of confirmed cases in the Republic of Cyprus were HCWs. Various reasons may explain this high rate of infection among HCWs, stressing the need for enhancement of preparedness through targeted interventions: limited awareness, particularly during the early phase of the epidemic; inadequate knowledge and training on personal protective measures; insufficient reporting of patients’ recent exposures and epidemiological history, as well as the high rate of asymptomatic patients that may have unintentionally transmitted [30].

An additional finding from our analyses is the notable proportion of imported cases (17.1%), the majority of which were repatriated Cypriot citizens and mainly detected during the initial weeks of the epidemic. In conjunction with recent reports of high rates of positive cases among repatriated passengers [31], this lends support to the relevance of early implementation of travel-associated restrictions and containment strategies, including travel bans, testing, and quarantine of travelers [32].

Our SAR estimate (12%) was similar to those in Hong Kong (11.7%) [16] (11.7%), and to estimates from Germany (SAR: 10% among household contacts) [33]. However, they were lower than those reported in earlier Chinese studies (SAR: 35% and 15%) [14]. The comparatively low SAR in the Republic of Cyprus might be attributable to the timely application of strict preventive measures in the community. Notably, enhanced nonpharmaceutical interventions, particularly case isolation, have been shown to substantially shorten the mean serial intervals (number of days between the symptom-onset date of the infector and that of the infectee among pairs) of COVID-19 in China within a month [34].

Our analyses have some limitations. First, we performed complete case analysis, thus excluding observations with missing data. Bias is likely in analyses with more than 10% missingness and if more than 40% data are missing in important variables then results should only be considered to be hypothesis generating [35]. In our analyses, although we retrieved up to 37% of missing data (i.e., smoking status), the multivariable model included variables with up to 12% missing information. Second, the testing rate in the Republic of Cyprus was very high and most symptomatic cases were probably diagnosed [36]. However, under-ascertainment of the true number of infections is likely, which did not allow us to estimate the infection fatality risk. Finally, we used surveillance data and significant information (e.g., laboratory data) that could explain some of the variability in multivariable models might have not been collected, as routinely performed in observational or interventional studies.

## 5. Conclusions

In conclusion, our analyses show that early implementation of public health interventions, including non-pharmaceutical measures and active contact tracing with extensive and targeted testing, proved to be a valuable combination of strategies to effectively control the COVID-19 outbreak in the Republic of Cyprus. Most characteristics of COVID-19 patients in the Republic of Cyprus confirm findings from other countries, including frequency of symptoms and risk factors for hospitalization and mortality. A high proportion of our cases were HCWs, underlining the need for targeted interventions to prevent SARS-CoV-2 transmission in healthcare settings.

## Figures and Tables

**Table 1:** Characteristics of laboratory-confirmed COVID-19 cases (*n* = 873) and deaths among COVID-19 cases (*n* = 20). Republic of Cyprus, 9 March–3 May 2020.

Characteristics	Cases (*n* = 873)		Alive (*n* = 853)		Deaths (*n* = 20)		CFR (95%CI)	*P* *
	*n*	%	*n*	%	*n*	%		
Origin of infection								
Imported	125	17.1	122	17.1	3	17.6	2.4 (0.5–6.9)	1
Locally acquired	604	82.9	590	82.9	14	82.4	2.3 (1.3–3.9)	
Missing	144		141		3			
Sex								
Male	437	50.1	421	49.4	16	80.0	3.7 (2.1–5.9)	**0.011**
Female	436	49.9	432	50.6	4	20.0	0.9 (0.3–2.3)	
Age group (years)								
0–9	27	3.1	27	3.2	0	0.0	0	**<0.001**
10–19	34	3.9	34	4.0	0	0.0	0	
20–29	112	12.8	112	13.1	0	0.0	0	
30–39	173	19.8	173	20.3	0	0.0	0	
40–49	152	17.4	151	17.7	1	5.0	0.7 (0–3.6)	
50–59	159	18.2	158	18.5	1	5.0	0.6 (0–3.5)	
60–69	111	12.7	105	12.3	6	30.0	5.4 (2–11.4)	
70–79	80	9.2	72	8.4	8	40.0	10 (4.4–18.8)	
80+	25	2.9	21	2.5	4	20.0	16 (4.5–36.1)	
Mean ± SD (Median; (IQR))	46 ± 18.8 (46; (32–59))		45 ± 18.4 (45; (32–59))		73 ± 10.2 (76; (67–79))			**<0.001**
District								
Famagusta	41	4.7	38	4.5	3	15.0	7.3 (1.5– 9.9)	0.067
Larnaca	228	26.1	222	26.0	6	30.0	2.6 (1.0–5.6)	
Limassol	97	11.1	95	11.1	2	10.0	2.1 (0.3–7.3)	
Nicosia	334	38.3	331	38.8	3	15.0	0.9 (0.2–2.6)	
Pafos	154	17.6	148	17.4	6	30.0	3.9 (1.4–8.3)	
Other (Unknown, abroad, British bases)	19	2.2	19	2.2	0	0.0	0	
Healthcare–worker								
No	686	78.6	666	78.1	20	100.0	2.9 (1.8–4.5)	**0.011**
Yes	187	21.4	187	21.9	0	0	0	
Physician	39	20.9	39	20.9	0	0	0	NC
Nurse	94	50.3	94	50.3	0	0	0	NC
Other healthcare worker	13	7	13	7	0	0	0	NC
Auxiliary staff	41	21.9	41	21.9	0	0	0	NC
Smoking status								
No	444	80.9	439	81.1	5	62.5	1.1 (0.4–2.6)	0.183
Yes	105	19.1	102	18.9	3	37.5	2.9 (0.6 –8.1)	
Missing	324		312		12			
Symptoms at diagnosis								
No	265	30.8	264	31.4	1	5.6	0.4 (0–2.1)	**0.018**
Yes	594	69.2	577	68.6	17	94.4	2.9 (1.7–4.5)	
≥3 symptoms	240	27.9	234	27.8	6	33.3	2.5 (0.9–5.4)	NC
Cough	303	51.0	293	35.2	10	58.8	3.3 (1.6–6.0)	NC
Fever	276	46.5	268	32.3	8	47.1	2.9 (1.3–5.6)	NC
Sore throat	153	25.8	151	18.2	2	12.5	1.3 (0.2–4.6)	NC
Myalgia	194	32.7	191	23	3	18.8	1.6 (0.3–4.5)	NC
Shortness of breath/respiratory distress	104	17.5	98	12	6	37.5	5.8 (2.1–12.1)	NC
Anosmia	122	20.5	122	16.3	0	0	0	NC
Diarrhea	94	15.8	92	11.1	2	11.8	2.1 (0.3–7.5)	NC
Missing	14		12		2			
Underlying health conditions								
No	450	58.8	447	60.0	3	15.0	0.7 (0–1.9)	**<0.001**
Yes	315	41.2	298	40.0	17	85.0	5.4 (3.2–8.5)	
≥2 conditions	98	12.8	86	11.5	12	60.0	12.2 (6.5–20.4)	NC
Diabetes	68	8.9	61	8.2	7	35.0	10.3 (4.2–20.1)	NC
Hypertension	124	16.4	119	16.1	5	25.0	4 (1.3–9.2)	NC
Heart disease (excluding Hypertension)	61	8	52	7.0	9	45.0	14.8 (7.0–26.2)	NC
Chronic kidney disease	14	1.9	9	1.2	5	25.0	35.7 (12.8–64.9)	NC
Chronic respiratory disease, excluding asthma	15	2.2	12	1.8	3	15.0	20 (4.3–48.1)	NC
Chronic liver disease	7	0.9	4	0.6	3	15.0	42.9 (9.9–81.6)	NC
Immunosuppression/HIV	15	2	15	2.1	0	0.0	0	NC
Cancer	21	2.7	18	2.4	3	15.0	14.3 (3.0–36.3)	NC
Neuromuscular disorder, chronic neurological	11	1.4	11	1.5	0	0.0	0	NC
Rheumatic diseases including arthritis	8	1	8	1.1	0	0.0	0	NC
Asthma	26	3.4	26	3.5	0	0.0	0	NC
Other endocrine disorder (excluding Diabetes)	22	2.9	22	3.0	0	0.0	0	NC
Missing	108		108		0			
Hospitalization								
No	706	80.9	706	82.8	0	0.0	0	**<0.001**
Yes	167	19.1	147	17.2	20	100.0	12 (7.5–17.9)	
ICU admission								
No	841	96.3	834	97.8	7	35.0	0.8 (0.3–1.7)	**<0.001**
Yes	32	3.7	19	2.2	13	65.0	40.6 (23.7–59.4)	
Intubation								
No	846	96.9	836	98.0	10	50.0	1.2 (0.6–2.2)	**<0.001**
Yes	27	3.1	17	2.0	10	50.0	37.0 (19.4–57.6)	

CFR: case-fatality ratio; CI: confidence interval; SD: standard deviation; IQR: interquartile range; NC: not calculated. * *p* values <0.05 are indicated in bold font.

**Table 2:** Characteristics of cases by hospitalization status, and factors associated with hospitalization (univariate and multivariable analysis), Republic of Cyprus 9 March–3 May 2020 ^1^.

Characteristics	Hospitalized (*n* = 167)		Not Hospitalized (*n* = 706)		OR (95%CI)	aOR (95%CI)
	*n*	%	*n*	%		
Male	110	65.9	327	46.3	**2.2 (1.6–3.2)**	**3.0 (2.0–4.7)**
Age group						
0–9	4	2.4	23	3.3	Ref	**1.6 (1.4–1.8) ^2^**
10–19	1	0.6	33	4.7	0.2 (0.0–1.7)	
20–29	7	4.2	105	14.9	0.4 (0.1–1.4)	
30–39	9	5.4	164	23.2	0.3 (0.1–1.1)	
40–49	21	12.6	131	18.6	0.9 (0.3–2.9)	
50–59	33	19.8	126	17.8	1.5 (0.5–4.7)	
60–69	39	23.4	72	10.2	**3.1 (1.0–9.7)**	
70–79	35	21	45	6.4	**4.5 (1.4–14.1)**	
80+	18	10.8	7	1	**14.8 (3.7–58.5)**	
Smoking status (yes)	14	17.5	91	19.4	0.9 (0.5–1.6)	NI
Symptoms at diagnosis (yes)	144	88.9	450	64.6	**4.4 (2.6–7.3)**	**6.1 (3.2–11.5)**
Cough (yes)	90	57.3	213	30.8	**3.0 (2.1–4.3)**	NI
Fever (yes)	87	55.4	189	27.4	**3.3 (2.3–4.7)**	NI
Sore throat (yes)	26	16.7	127	18.5	0.9 (0.6–1.4)	NI
Myalgia (yes)	53	34.2	141	20.4	**2.0 (1.4–3.0)**	NI
Shortness of breath/respiratory distress (yes)	45	29.6	59	8.7	**4.4 (2.9–6.9)**	NI
Anosmia (yes)	16	12.8	106	16.7	0.7 (0.4–1.3)	NI
Diarrhea (yes)	21	13.5	73	10.6	1.3 (0.8–2.2)	NI
Underlying health conditions (yes)	98	69.5	217	34.8	**4.3 (2.9–6.3)**	**2.1 (1.3–3.3)**
Diabetes (yes)	34	24.1	34	5.4	**5.5 (3.3–9.3)**	NI
Hypertension (yes)	44	31.4	80	12.9	**3.1 (2.0–4.7)**	NI
Heart disease (excluding Hypertension) (yes)	31	22.1	30	4.8	**5.6 (3.3–9.6)**	NI
Chronic kidney disease (yes)	10	7.2	4	0.6	**11.9 (3.7–38.5)**	NI
Chronic respiratory disease, excluding asthma (yes)	5	4.1	10	1.8	2.4 (0.8–7.0)	NI
Chronic liver disease (yes)	5	3.6	2	0.3	**11.4 (2.2–59.5)**	NI
Immunosuppression/HIV (yes)	4	2.9	11	1.8	1.6 (0.5–5.1)	NI
Cancer (yes)	9	6.4	12	1.9	**3.5 (1.4–8.4)**	NI
Neuromuscular disorder, chronic neurological (yes)	3	2.1	8	1.3	1.7 (0.4–6.4)	NI
Rheumatic diseases including arthritis (yes)	0	0	8	1.3	–	NI
Asthma (yes)	7	5	19	3	1.7 (0.1–4.0)	NI
Other endocrine disorder (excluding Diabetes) (yes)	2	1.4	20	3.2	0.4 (0.1–1.9)	
Death (yes)	20	12	0	0	–	NI

OR: odds ratio; CI: confidence interval; a: adjusted; NI: not included; ^1^ Significant associations (*p* < 0.05) are indicated in bold font ^2^ Age group treated as continuous variable.

**Table 3:** Total people screened and positive results by sex and age group—Targeted testing of employees in the public domain, in retail food and beverage services, in customer services, of the personnel of National Guard, and in nursing homes, Republic of Cyprus, 10–30 April 2020.

Age Groups (years)	Male			Female			Total		
	Screened	Positive		Screened	Positive		Screened	Positive	
	*N*	*n*	%	*N*	*n*	%	*N*	*n*	%
<20	104	0	0.00	43	0	0.00	153	0	0.00
20–24	388	1	0.26	444	0	0.00	884	1	0.11
25–29	922	1	0.11	920	1	0.11	1953	2	0.10
30–34	1213	3	0.25	850	0	0.00	2179	3	0.14
35–39	1351	2	0.15	965	1	0.10	2423	3	0.12
40–44	1379	3	0.22	1218	1	0.08	2696	4	0.15
45–49	1374	1	0.07	1281	5	0.39	2758	6	0.22
50–54	1229	3	0.24	1202	1	0.08	2516	4	0.16
55–59	1040	3	0.29	937	2	0.21	2033	5	0.25
60–64	619	0	0.00	598	2	0.33	1244	2	0.16
65+	220	0	0.00	133	0	0.00	359	0	0.00
Unknown	906	0	0.00	591	0	0.00	1598	0	0.00
Total	10,745	17	0.16	9182	13	0.14	20,796	30	0.14

## Supplementary Materials

The following are available online: https://www.mdpi.com/2077-0383/9/11/3598/s1, Figure S1: 14-day cumulative notification rate (per 100,000 population) in Municipality A, Municipality B and in total, Cyprus 9 March–3 May 2020, Table S1: Age-specific notification rate by sex, Cyprus 9 March–3 May 2020, Table S2: Characteristics of cases by recovery status, and factors associated with recovery status (univariate), Cyprus 9 March–3 May 2020.
