# Supplementary material for: Extensive Testing and Public Health Interventions for the Control of COVID-19 in the Republic of Cyprus between March and May 2020
Source: J Clin Med. 2020 Nov 8;9(11):3598. doi: 10.3390/jcm9113598 (PMC7695263; doi:10.3390/jcm9113598)
Supplement: Supplementary file 1 [file jcm-09-03598-s001.pdf]

Supplementary Materials: Extensive Testing and Public Health Interventions for the Control of COVID-19 in the Republic of Cyprus between March and May 2020

Annalisa Quattrocchi, Ioannis Mamais, Constantinos Tsioutis, Eirini Christaki, Costas Constantinou, Maria Koliou, Zoi-Dorothea Pana, Valentinos Silvestros, Fani Theophanous, Christos Haralambous, Androulla Stylianou, Sotiroula Sotiriou, Maria Athanasiadou, Theopisti Kyprianou, Anna Demetriou, Christiana A. Demetriou, Ourania Kolokotroni, Ioanna Gregoriou, Niki Paphitou, George Panos, Leontios Kostrikis, Peter Karayiannis, Georgios Petrikkos, Petros Agathangleou, George Mixides, Georgios Siakallis, Linos Hadjihannas, Lakis Palazis, Anna Vavlitou, Chrystalla Matsentidou-Timiliotou, Dimitris Koukios, Tonia Adamidi, Frangiskos Frangopoulos, Elizabeth Constantinou and Georgios Nikolopoulos

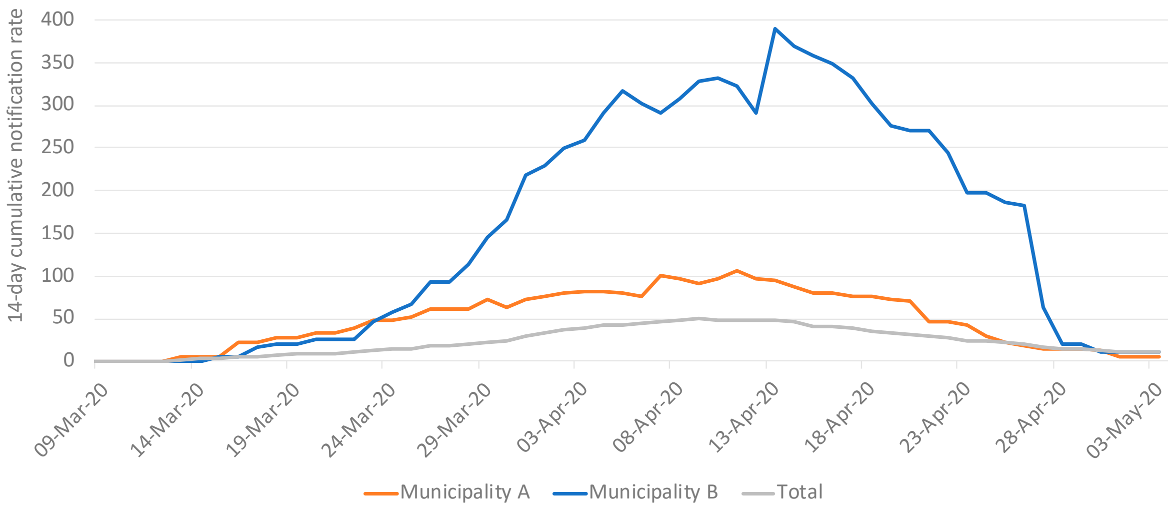

Figure S1. 14-day cumulative notification rate (per 100,000 population) in Municipality A, Municipality B and in total, Cyprus March 9th - May 3rd, 2020.

Table S1. Age-specific notification rate by sex, Cyprus 9 March–3 May 2020.

Age groups (years)	TOTAL			MALES			FEMALES		
	Cyprus Population [1]	Notified cases	Crude Age-specific Notofication Rate	Cyprus Population[1]	Notified cases	Crude Age-specific Notofication Rate	Cyprus Population[1]	Notified cases	Crude Age-specific Notofication Rate
Less than 5	46,630	14	30.02	24,016	5	20.82	22,614	9	39.80
From 5 to 9	49,104	13	26.47	24,966	7	28.04	24,138	6	24.86
From 10 to 14	45,470	15	32.99	23,200	11	47.41	22,270	4	17.96
From 15 to 19	48,856	19	38.89	24,429	8	32.75	24,427	11	45.03
From 20 to 24	63,295	46	72.68	30,018	21	69.96	33,277	25	75.13
From 25 to 29	74,578	66	88.50	36,530	33	90.34	38,048	33	86.73
From 30 to 34	73,209	83	113.37	35,955	45	125.16	37,254	38	102.00
From 35 to 39	66,650	90	135.03	32,678	46	140.77	33,972	44	129.52
From 40 to 44	56,158	67	119.31	27,240	37	135.83	28,918	30	103.74
From 45 to 49	54,054	85	157.25	26,325	41	155.75	27,729	44	158.68
From 50 to 54	53,871	78	144.79	26,614	34	127.75	27,257	44	161.43
From 55 to 59	54,546	81	148.50	26,958	37	137.25	27,588	44	159.49
From 60 to 64	48,366	58	119.92	23,846	25	104.84	24,520	33	134.58
From 65 to 69	45,193	53	117.27	21,966	31	141.13	23,227	22	94.72

From 70 to 74	36,574	42	114.84	17,152	17	99.11	19,422	25	128.72
From 75 to 79	27,332	38	139.03	12,590	22	174.74	14,742	16	108.53
80 and over	32,013	25	78.09	13,348	17	127.36	18,665	8	42.86
Total	875,899	873	99.67	427,831	437	102.14	448,068	436	97.31

[1] Data from Statistical Service of the Republic of Cyprus, 2018 [14].

Table S2. Characteristics of cases by recovery status, and factors associated with recovery status (univariate), Cyprus 9 March–3 May 2020.

**Characteristics**	**Recovered/cured (*n* = 396)**		**Not recovered/cured (*n* = 317)**		p [1]
	n	%	*n*	%	
Male	187	47.2	163	51.4	0.265
Age group					
0-9	9	2.3	9	2.8	
10-19	9	2.3	18	5.7	
20-29	52	13.1	35	11.0	
30-39	87	22.0	61	19.2	
40-49	68	17.2	60	18.9	0.439
50-59	79	19.9	58	18.3	
60-69	49	12.4	39	12.3	
70-79	34	8.6	27	8.5	
80+	9	2.3	10	3.2	
Symptoms at diagnosis (yes)	285	73.5	219	69.5	0.171
Cough (yes)	150	53.0	110	51.6	0.764
Fever (yes)	126	44.5	108	51.2	0.142
Sore throat (yes)	90	31.9	42	19.9	**0.003**
Myalgia (yes)	93	32.9	80	37.9	0.244
Shortness of breath/respiratory distress (yes)	52	19.1	40	19.2	0.975
Anosmia (yes)	53	23.2	60	32.3	**0.041**
Diarrhoea (yes)	52	18.4	33	15.6	0.412
Underlying health conditions (yes)	128	39.1	126	44.7	0.167
Diabetes (yes)	22	8.5	30	12.7	0.191
Hypertension (yes)	49	38.3	52	41.6	0.59
Heart disease (excluding Hypertension) (yes)	25	19.8	21	16.7	0.514
Chronic kidney disease (yes)	3	2.4	5	4.0	0.722
Chronic respiratory disease, excluding asthma (yes)	7	6.5	3	2.8	0.214
Chronic liver disease (yes)	2	1.6	1	0.8	1
Immunosuppression/HIV (yes)	7	5.7	8	6.4	0.828
Cancer (yes)	7	5.5	6	4.8	0.798
Neuromuscular disorder, chronic neurological (yes)	4	3.1	7	5.6	0.374
Rheumatic diseases including arthritis (yes)	5	3.9	2	1.6	0.447
Asthma (yes)	8	6.3	9	7.1	0.776
Other endocrine disorder (excluding Diabetes) (yes)	10	7.8	9	7.1	0.839
Hospitalised (yes)	64	16.2	61	19.2	0.282

[1] p values <0.05 are indicated in bold font.
